# Rediscovering Langhans' Fibrinoid Layer: A Forgotten Barrier at the Maternal–Fetal Interface

**DOI:** 10.30699/ijp.2025.2058811.3446

**Published:** 2025-11-11

**Authors:** Rohini Motwani, Seetu Palo, Mishu Mangla

**Affiliations:** 1 *Department of Anatomy, All India Institute of Medical Sciences, Bibinagar, Telangana, India*; 2 *Department of Pathology and Laboratory Medicine, All India Institute of Medical Sciences, Bibinagar, Telangana, India*; 3 *Department of Obstetrics and Gynaecology, All India Institute of Medical Sciences, Bibinagar, Hyderabad, India*

**Dear Editor,**

In placental histopathology, the Langhans’ fibrinoid layer occupies a unique but often overlooked position. Although described more than a century ago and featured in classic medical texts, it has gradually faded from modern research and is inconsistently defined in contemporary literature. Langhans’ fibrinoid remains one of the most underappreciated and ambiguously described placental structures. In this communication, we revisit its historical origins, anatomical ambiguity, and potential clinical relevance, and argue for its clear redefinition in current placental studies and diagnostics.

Theodor Langhans first described, in 1877, an amorphous, acellular, eosinophilic deposit located between the cytotrophoblasts of anchoring villi and the maternal decidua basalis during early placental development (1). Termed “fibrinoid” because of its fibrin-like appearance on hematoxylin and eosin staining, this zone was depicted as a distinct band in early 20th-century literature but without clear separation from neighboring structures (1). With advances in histological and imaging techniques, other fibrinoid layers—most notably Nitabuch’s layer at the decidual–trophoblast interface and Rohr’s fibrinoid beneath the syncytiotrophoblast—were described. These newer definitions created terminological overlap and led to the gradual abandonment of Langhans’ original term (2). Subchorionic fibrinoid, a fibrin-rich deposit beneath the chorionic plate, is often conflated with Langhans’ fibrinoid but is histologically distinct.

As Nitabuch’s layer gained diagnostic significance in placenta accreta spectrum disorders, Langhans’ fibrinoid was relegated to generalized references under “fibrinoid” or “fibrin deposition.” However, emerging evidence suggests that Langhans’ fibrinoid plays important roles in placental physiology, including immunological barrier function, mechanical stabilization, and modulation of pathological processes (3). In preeclampsia, it often demonstrates thickening and disorganization in response to defective trophoblastic invasion and impaired spiral artery remodeling. Uteroplacental ischemia and oxidative stress trigger trophoblast apoptosis and extracellular matrix protein deposition, leading to expansion of Langhans’ fibrinoid and disruption of the maternal–fetal interface (4). Similar though subtler alterations are noted in fetal growth restriction, with thickened and irregular fibrinoid infiltrated by apoptotic trophoblasts, suggesting a chronic hypoxic environment (5). Despite clinical recognition, no universally accepted morphometric scale or consensus definition exists to distinguish normal from pathological Langhans’ fibrinoid.

In inflammatory and immune-mediated conditions, such as chronic intervillositis of unknown etiology and massive perivillous fibrin deposition (MPFD), abnormal expansion of fibrinoid reflects sustained trophoblast injury and impaired immune tolerance (6,7). MPFD, in particular, involves extensive fibrinoid encasing chorionic villi, disrupting maternal–fetal exchange and mimicking infarction, with frequent associations to recurrent pregnancy loss and intrauterine fetal demise (7,8). Even in metabolic disorders such as diabetes mellitus, Langhans’ fibrinoid thickens in response to hyperglycemia-induced oxidative stress and endothelial dysfunction, contributing to placental inefficiency and altered fetal growth patterns (9,10).

Comparative descriptions of Langhans’, Nitabuch’s, and Rohr’s fibrinoid layers exist (Table 1, Figure 2), yet diagnostic ambiguity persists due to inconsistent terminology and the generalized use of “fibrinoid” to describe eosinophilic, amorphous deposits in different placental zones. This lack of standardized nomenclature and diagnostic criteria complicates recognition of pathological fibrinoid changes in disorders such as preeclampsia, FGR, and stillbirth. Establishing consensus definitions, supported by molecular markers, could improve diagnostic reproducibility and deepen understanding of placental pathophysiology. To this end, we propose targeted strategies in education, diagnostic terminology, and research, summarized in Table 2.

In conclusion, Langhans’ fibrinoid should not be dismissed as a historical curiosity. Rather, it represents a specialized matrix niche essential for balanced trophoblast function, immune tolerance, and mechanical stability at the maternal–fetal interface. We advocate for its clear redefinition and reintegration into teaching curricula and research frameworks. Rediscovering this forgotten barrier may provide valuable insights into placental development and disease.

**Fig. 1:**
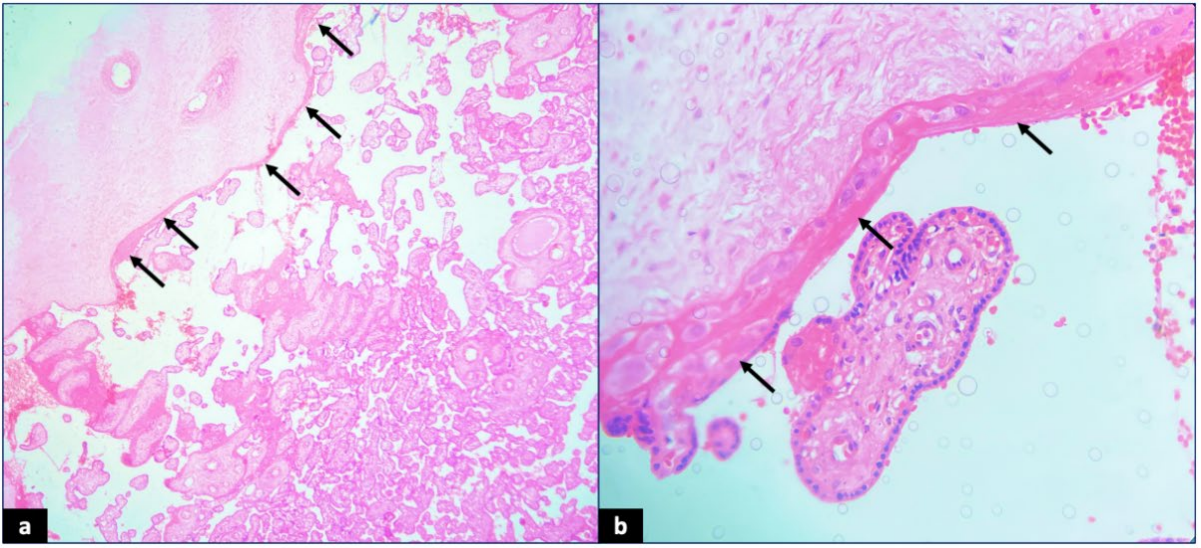
Langhans’ fibrinoid layer (arrow) with adjacent chorionic plate: (a) Low-power view [40x, Hematoxylin and eosin]; (b) High-power view [400x, Hematoxylin and eosin]

**Fig 2:**
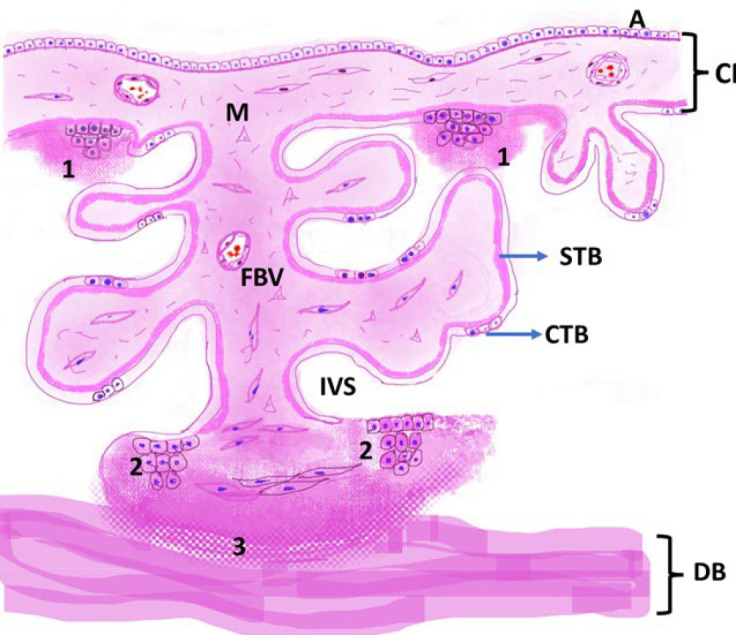
Schematic representation of Fibrinoid layers of placenta (created by one of the authors, RM)-  (1) Langhans' stria or subchorial fibrinoid comprises the fibrinoid lining the intervillous surface (IVS) of the chorionic plate; (2) Rohr's stria or Rohr’s fibrinoid, a superficial layer of fibrinoid covering the intervillous surface (IVS) of the basal plate (BP); (3) Nitabuch's stria or Nitabuch's layer, fibrinoid located deeper in the basal plate, embedding maternal and fetal cells. STB: Syncytiotrophoblast, CTB: Cytotrophoblast, FBVs: Fetal Blood Vessels.

**Table 1:** Distinguishing features and pathological associations of the three fibrin layers of placenta

Features	LANGHANS’ FIBRINOID	NITABUCH’S LAYER	ROHR’S FIBRINOID
Location	Beneath the chorionic plate (subchorionic region)	At decidual-trophoblast interface	At the base of anchoring villi
Composition	Fibrin, trophoblast debris, extracellular matrix	Fibrin, decidual cells, trophoblast remnants	Fibrin, perivillous fibrinoid, immune cell infiltrates
Function	Immunological barrier and mechanical stabilisation	Anchors placenta to decidua, prevents invasion into myometrium	Structural support and acts as a barrier beneath anchoring villi at the maternal–fetal interface.
Pathological Relevance	Increased thickness suggests maternal vascular malperfusion, chronic inflammation	Absence/disruption is suggestive of placenta accreta spectrum	Excessive deposition is suggestive of hypoxia, villitis or infections
Clinical Associations	Fetal growth restriction, preeclampsia, chronic intervillositis	Placenta accreta syndrome, Placenta previa	Recurrent pregnancy loss, villitis of unknown etiology

**Table 2:** Proposed educational and research initiatives to reintegrate Langhans’ fibrinoid into placental pathology discourse.

Reintroducing eponymous layers in histological teaching	Langhans' fibrinoid should be taught alongside Nitabuch's and Rohr's layers in medical and pathology curricula to improve anatomical precision at the maternal-fetal interface. High-resolution annotated virtual slides and interactive 3D reconstructions of the maternal-fetal interface highlighting Langhans’ fibrinoid, alongside Nitabuch’s and Rohr’s layers, may be used as teaching-learning methods.
Integrate classical nomenclature and modern classifications	To avoid losing spatial and functional context in pathology reporting, align conventional words (for example, Langhans' fibrinoid) with biochemical classifications (fibrin-type, matrix-type).
Encourage histopathological redescription in clinical practice	Examine archived histology slides from diagnosed conditions such as preeclampsia, placenta accreta spectrum, and growth‑restricted pregnancies to systematically document the presence, absence, thickness, and integrity or modification of Langhans' fibrinoid so that to evaluate its diagnostic and prognostic utility.
Promote targeted research	Investigate Langhans' fibrinoid's cellular origin, molecular composition, and immunomodulatory activities utilising technologies such as immunohistochemistry, single‑cell RNA‑seq, proteomics, and spatial transcriptomics. Hence, we can profile the cellular and molecular milieu within Langhans’ fibrinoid, differentiating it from Nitabuch’s and Rohr’s zones.
Standardize terminology in diagnostic criteria and encourage inclusion in placental reporting templates	Include Langhans’ fibrinoid as a recognizable histoanatomical structure in future consensus statements (e.g., Amsterdam Placental Workshop criteria) to aid diagnostic clarity, along with its inclusion in standard placental pathology reporting templates.
